# Review of cone beam computed tomography based online adaptive radiotherapy: current trend and future direction

**DOI:** 10.1186/s13014-023-02340-2

**Published:** 2023-09-02

**Authors:** Hefei Liu, David Schaal, Heather Curry, Ryan Clark, Anthony Magliari, Patrick Kupelian, Deepak Khuntia, Sushil Beriwal

**Affiliations:** 1 Present Address: Department of Radiation Oncology, University of Pennsylvania, Philadelphia, USA; 2 Varian Medical Systems Inc, Palo Alto, CA USA; 3 Allegheny Health Network Cancer Institute, Pittsburgh, PA USA

**Keywords:** Cone-beam computed tomography (CBCT), Adaptive radiotherapy (ART), Ethos™

## Abstract

Adaptive radiotherapy (ART) was introduced in the late 1990s to improve the accuracy and efficiency of therapy and minimize radiation-induced toxicities. ART combines multiple tools for imaging, assessing the need for adaptation, treatment planning, quality assurance, and has been utilized to monitor inter- or intra-fraction anatomical variations of the target and organs-at-risk (OARs). Ethos™ (Varian Medical Systems, Palo Alto, CA), a cone beam computed tomography (CBCT) based radiotherapy treatment system that uses artificial intelligence (AI) and machine learning to perform ART, was introduced in 2020. Since then, numerous studies have been done to examine the potential benefits of Ethos™ CBCT-guided ART compared to non-adaptive radiotherapy. This review will explore the current trends of Ethos™, including improved CBCT image quality, a feasible clinical workflow, daily automated contouring and treatment planning, and motion management. Nevertheless, evidence of clinical improvements with the use of Ethos™ are limited and is currently under investigation via clinical trials.

## Introduction

For the majority of courses of external beam radiotherapy today, a typical process begins with a single treatment plan generated at the simulation step and carried through the entire course [1]. Given time and resource constraints, these plans are rarely modified over the course of treatment despite the internal changes within the patient that may lead to errors in dose placement, suboptimal disease responses, and avoidable radiation-induced toxicities. Adaptive radiotherapy (ART) was introduced in the late 1990s as a “closed-loop radiation treatment process where the treatment plan can be modified using a systematic feedback of measurements” [2, 3]. Its primary goal has been to improve the accuracy and efficiency of therapy and minimize radiation-induced toxicities. By 2010, ART was widely described within the radiation oncology literature [4, 5]. ART combines multiple tools for imaging, assessing the need for adaptation, treatment planning, quality assurance, and has been utilized to monitor inter- or intra-fraction anatomical variations of the target and organs-at-risk (OARs). These tools can enable dose escalation or maintaining coverage of target doses while reducing doses to OARs [3, 6–9].

The implementation of ART is categorized into three major classes. (1) Offline ART, in which scheduled imaging between fractions is used to detect systematic and progressive changes that occur during the treatment course. (2) Online ART entails the adjustment of treatment plans prior to radiation delivery to account for inter-fraction changes, both temporal and stochastic, while the patient remains in the treatment position. (3) Real-time ART, which accounts for intra-fractional variations and allows for automatic plan adjustments during radiation delivery without manual intervention [3]. Both offline [6, 10–13] and online [14–23] ART has shown improvements in target coverage and OAR sparing in cancers of the prostate, head and neck, lung, abdomen, and pelvis. Executing ART requires adequate information for target and OAR delineation, accurate dose calculation, and sufficient image quality [3].

Given rapid and recent advancements in online ART technology, most commonly delivered either with linear accelerators combined with onboard magnetic resonance imaging (“MRI-Linac”) or CBCT-guided systems, a review that describes the technical and clinical state of the art is necessary. This comprehensive review will focus on published and presented data on CBCT-guided online ART with the Ethos™ system. We also evaluate the feasibility of applying this system to a greater clinical context and discuss future directions. We located peer-reviewed articles and abstracts on the topic of CBCT-guided online ART published from 2019, which was the first year that Ethos was under investigation, to present day. A search of following terms were conducted in PubMed: ““CBCT-guided” OR “CT-Guided” AND “real-time adaptive” OR “online adaptive” OR “ontable adaptive” OR “Ethos”. We excluded publications regarding proton therapy, brachytherapy, MR-Linac, offline ART, and CT-based ART in which CT is not an integrated part of the treatment system (e.g., CT on rails).

### CBCT-based online ART

Before each treatment, the process of CBCT-guided ART begins with the use of a cone shaped X-ray beam where the kilovoltage (kV) source and a flat panel detector rotates around a patient on the treatment table [24, 25]. The acquired CT image is then automatically segmented into various organs and bony structures [26]. Based on this delineation of the day’s anatomy, the system generates a preview of the dose distribution for OARs and the disease target from a prioritized list of clinical goals, followed by creation of multiple deliverable treatment plans [26]. At this time, the best plan deemed by the radiation oncologist was selected as the reference plan and approved through a quality assurance (QA) protocol [26]. Finally, treatment is delivered to the patient, and the process is repeated prior to subsequent treatments.

Conventional CBCT has been widely used for positioning of patients during ART, but has significantly inferior image quality due to increased radiation scatter compared to traditional fan-beam (planning) CT [27]. New image reconstruction algorithms such as iterative CBCT (iCBCT) have enhanced the overall quality CBCT image generation to create more accurate CBCT-based image-guided radiotherapy (IGRT) [27, 28]. Ethos™ (Varian Medical Systems, Palo Alto, CA), a radiotherapy treatment system that uses artificial intelligence (AI) and machine learning to perform ART, was introduced in 2020. The system integrates iCBCT [29], and provides a highly efficient adaptive workflow while the patient is on the treatment couch, allowing a physician to select either the reference plan or the adapted treatment plan within a typical 15–25 min scheduled time slot [29].

### kV CBCT image quality

High-quality images that are obtained quickly are necessary for auto-segmentation and on-table adaption during a short treatment time slot. Cai et al. investigated the image quality of kV CBCT on Halcyon™ 2.0, which the Ethos™ kV CBCT was built upon [30]. The kV CBCT can rapidly acquire high quality images with iterative reconstruction that yield high contrast-to-noise ratio (CNR) [30]. In addition, the fast gantry motion allows for single breath-holding imaging, which can minimize artifacts and provide potential for on-table structure delineation [30]. Schiff et al. also reported, the image quality of single breath-hold is acceptable for ART with Ethos™, as the treating physician or medical physicist did not reject any images due to poor quality [31, 32]. However, one limitation of kV CBCT is the narrowfield-of-view (FoV) which is overcome by fusing the daily kV CBCT with the planning CT and deformation as its registration [34].

**Fig. 1:**
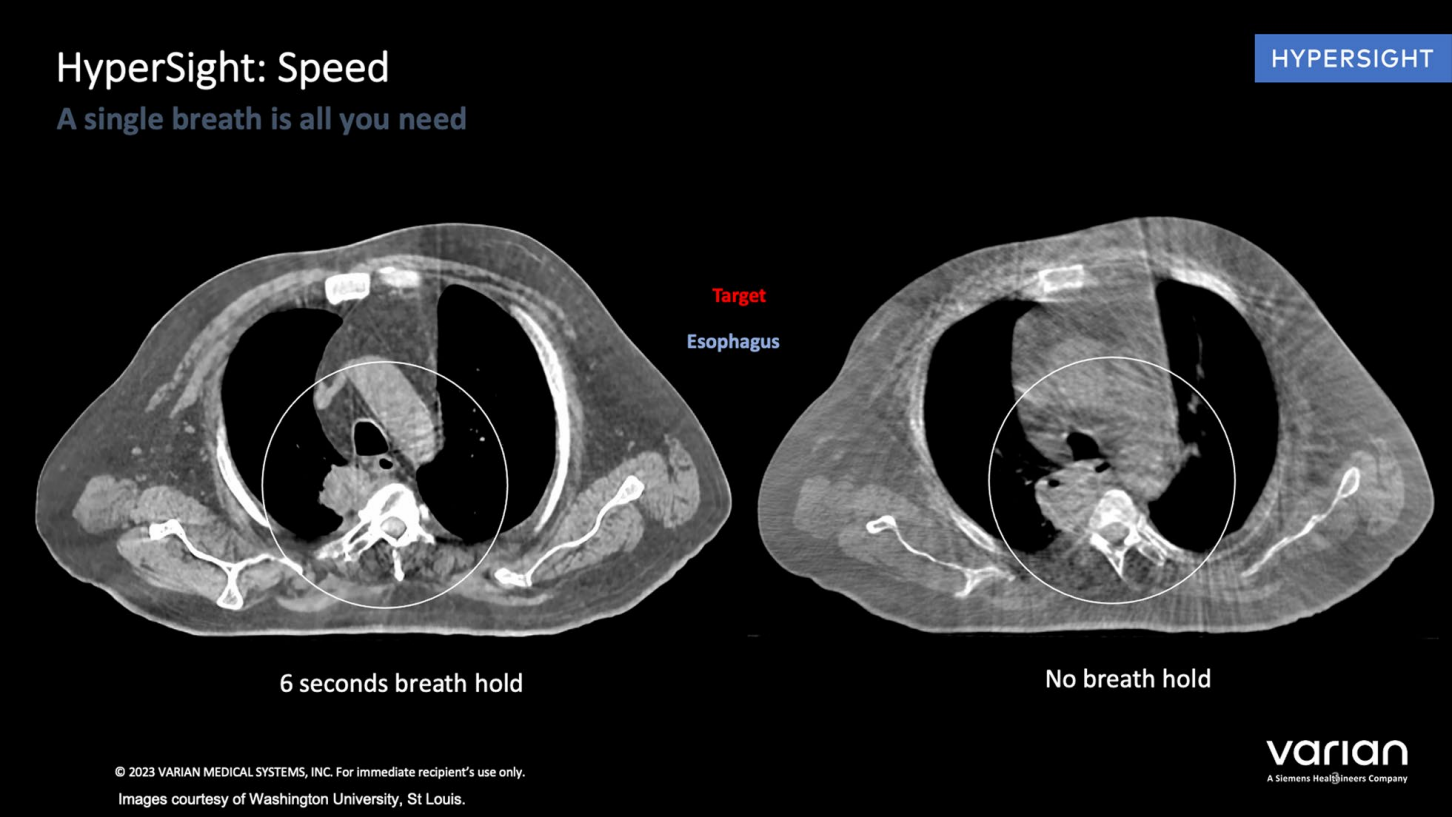
HyperSight™ CBCT technology captures larger images with better contrast within only 6 s, which allows tumors that move with respiration to comfortably perform a single breath-hold for the machine to obtain high-quality images needed for daily treatment planning and delivery

This limitation can be also be overcome by the new HyperSight™ CBCT technology which has novel features such as extended FoV up to 70 cm, as well as an advanced reconstruction techniques for improved image quality [33, 35, 36]. Also, it captures images in less than 6 s, which is 10 times faster than a conventional linear-accelerator-based imaging system (Fig. 1) [35, 37, 38]. This allows tumors that move with respiration to comfortably perform a single breath-hold for the machine to obtain high-quality images needed for daily treatment planning and delivery. It also includes the ability to perform dose calculation directly on the native images. The relevance of this advancement in ontable imaging to CBCT-guided ART is clear; clearer, high-contrast images will improve contouring, speed up the generation of adaptive plans, and enhance confidence in adaptive plan quality.,.

### Feasibility of CBCT-based online ART in normal clinical workflow

**Fig. 2:**
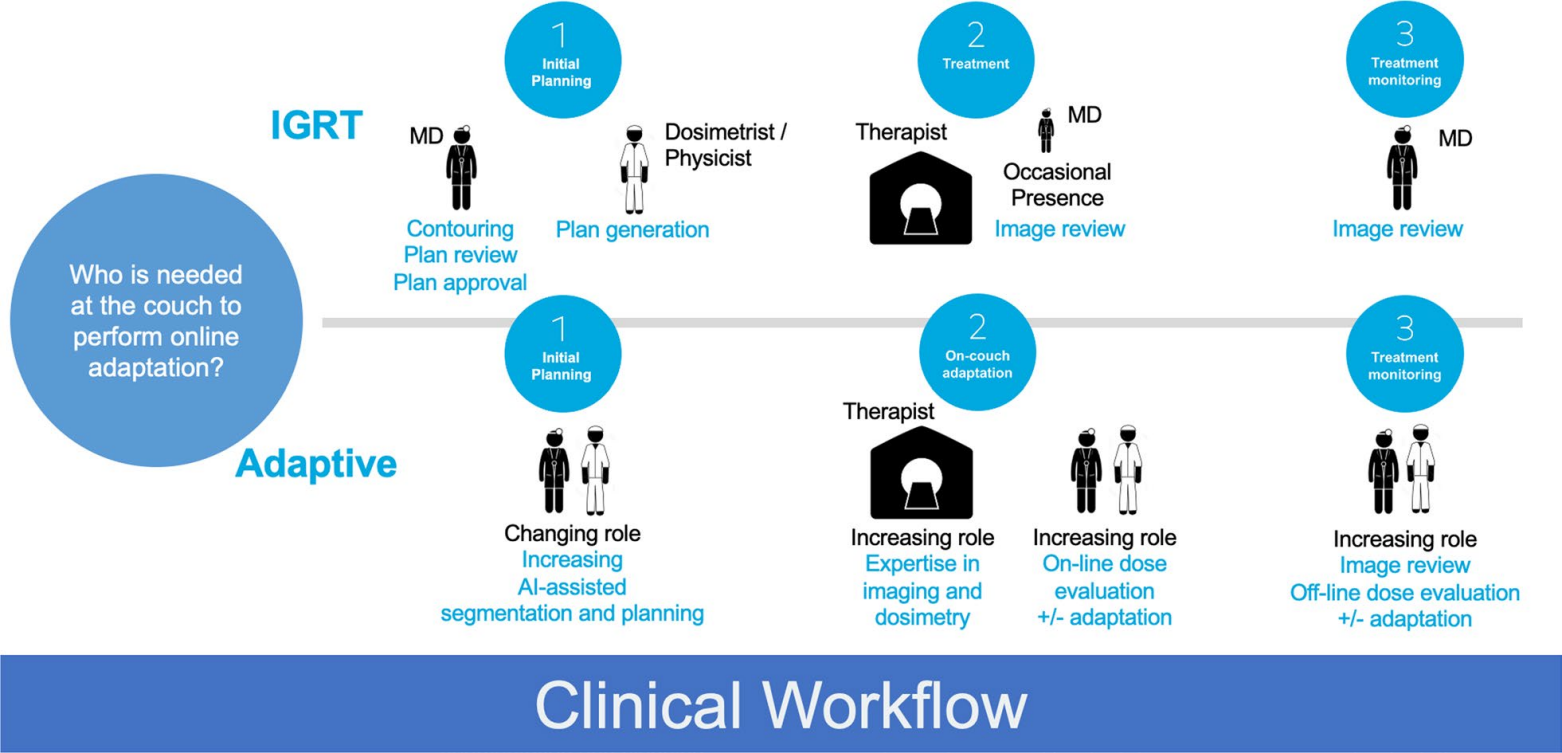
Workflow comparison between IGRT and adaptive radiotherapy

A typical appointment slot in a ARTtreatment session is generally reserved for 15- 30 min, thought the range could be larger. A large portion of the time will be used for precise positioning of the patient, followed by radiation delivery. The time to complete the adaptive component of the workflow, which is defined as the ART procedural time, includes additional steps between the collection of the CBCT and the first beam on. These steps include an auto-segmentation process, reviewing and optional editing of the autocontours, plan re-optimization, dose calculation, and a quality-assurance check (Fig. 2) [39, 40]. Often, clinician presence is required to assess and adjust online autocontouring and perform plan review [41]. Therefore, the introduction of ART has raised concerns about adding potentially time-consuming steps and clinician involvement into an already time-constrained workflow.

The ART procedural time heavily depends on the disease site (Table 1). For example, the reported Ethos™ ART procedural time on average ranged from 10 to 12 min for prostate cancer [42, 43]. The median overall planning time was mostly dependent on the number of fields (intensity-modulated radiotherapy [IMRT]) and arcs (volumetric modulated arc therapy [VMAT]) that were used: 2.6 min for 7 field IMRT; 3.1 min for 9 field IMRT; 3.4 min for 12 field IMRT; 13.2 min for 2 arc VMAT; and 14 min for 3 arc VMAT [36]. For complex prostate stereotactic body radiotherapy (SBRT), the procedural time is significantly longer [44].

**Table 1:** 17 published articles of dosimetric studies of CBCT-guided ART

Disease site	Article	Number of patients	Adapted fractions evaluated	ART procedural time	Outcomes
Abdominal oligometastases	Schiff et al. [31]	8	40	Average 22.59 min	30/40 fractions had OAR constraint violation under no adaption, compared to only 2/40 under daily adaptation Improved GTV V100% and D95% in 25/40 and 20/40 fractions, respectively
Breast	Montalvo et al. [48]	22	106/110	Average 15 min	PTV and CTV coverage improved significantly after adaption No difference in OAR coverage
Breast (prone)	Yoon et al. [67]	8	128	–	Daily CBCT showed key dose parameters vary significantly for patients undergoing tangential prone breast RT
Bladder	Åström et al. [33]	16	297/512	Median 13.9 min	Median 33.9% volume reduction in primary PTV volume Median 18.8% reduction in bowel bag V_45Gy_ Median 70.7% reduction in rectum V_50Gy_
Cervix	Branco et al. [34]	15	75/75	Average 16.8 min	CTV D_99%_ for adapted plans comparable to non-adapted plans Bowel V_45Gy_ and V_40Gy_ decreased on average by 87.6 and 109.4 cc, respectively Bladder and rectum D_50%_ decreased by 37.7% and 35.8%, respectively
Cervix and Rectum	Yock et al. [45]	28 (13 cervix, 15 rectum)	149 (cervical) and 162 (rectal)	Average 24.4 min (cervical) and 9.2 min (rectal)	Dosimetric benefits can be achieved with CBCT-based online ART Procedural time spent on online ART is amenable to conventional appointment slots
Rectum	de Jong et al. [25, 63]	20	300	Average 26 min	Target coverage were similar between adapted and non-adapted plans Normal tissue V_95%_ reduced from 642 to 237 cc with online ART Median difference for bowel bag V_15Gy_ was -126 cc (short course RT) and -62 cc (long course RT) Median difference for bladder V_15Gy_ was 26% (long course RT) Median difference for bladder V_95%_ was -8% (short course RT)
Head and Neck	Yoon et al. [47]	5	–	Average 19 min and 34 s	Adapted plan improved OAR sparing, while maintaining similar therapeutic dose to target
Lung	Mao et al. [55]	10	297	–	Improve average target coverage and planning target volume minimum doses Reduced average upper dose constraints from online ART
Pancreas	Schiff et al. [41]	8	40	Average 36.28 min	OAR and target dosimetry improved with online ART in 100% of fractions 39/40 fractions met all OAR constraints under daily adaptation, compared to 0/40 under no adaptation
Pelvis (Anal, Rectal, and Prostate)	Calmels et al. [39]	60 total (20 each)	–	Median 35–55 min	Automated treatment planning system implemented by online ART generated plans to comparable to manually generated plans
Pelvis (bladder, rectum, anal, and prostate)	Sibolt et al. [40]	39 (only 5 reported)	–	Average 17.6 min	Adapted plan was superior in 88% of cases 42% median primary PTV reduction in treated bladder patients and 24–30% V45Gy reduction to the bowel cavity compared to non-ART
Prostate	Byrne et al. [38]	18	182	Average 19 min	Adaptive plan was preferred in 95% of fractions Adaptive plan met more goals than scheduled plan in 78% of fractions Significant dosimetric improvements
Prostate	De Roover et al. [59]	20	–	–	Online ART resulted in increased seminal vesicle PTV coverage and reduced dose to the bladder and urethra, but increased dose to the rectum
Prostate	Moazzezi et al. [54]	25	250	–	96% of fractions required minor auto-segmentation edits Adaptation improved CTV D98% by 2.9% Adaptation reduced bladder and rectum V90% by 13.1% and 6.5%, respectively
Prostate	Morgan et al. [36]	20	248	Average 10.7 min	Online adaptive radiotherapy decreased PTV margins Adapted plans achieved significant reductions in rectum V40Gy and V65Gy
Prostate	Zwart et al. [37]	11	220	Average 11.9 min	PTV coverage increased for the adapted plan compared to the scheduled plan Adapted plans were more likely to meet Bladder and rectum V60Gy constraints

For abdominal and pelvic malignancies, which included reports on the pancreas, liver, bile duct, retroperitoneum, rectum, anus, and abdominal oligometastases, the average procedural time ranged from 17 to 36 min [31, 45–47]. The longer procedural time could be explained by the normal peristaltic motion of the gastrointestinal (GI) tract, as well as daily differences in bladder filling and stool burden, leading to a higher tendency for abdominal organs to shift [48]. Therefore, more time is likely required for contouring edits of the PTV and OARs by the treating physician. Specifically for locally advanced pancreatic cancer (LAPC), the emphasis on dose-escalated radiotherapy to improve local control and survival outcomes can further increase the ART procedural time, with OAR contouring being the most time-consuming step [47]. ART for bladder cancer (procedural time ranging from 14 to 32 min) [39, 49, 50] and cervical cancer (16 to 24 min) [40, 51] follows a similar logic with bladder filling and stool burden, while lung malignancies (15 min) [52] is heavily dependent on respiratory motion, all of which would lead to relatively longer procedural time (Tables 1, 2).

**Table 2:** 10 published abstracts of dosimetric studies of CBCT-guided ART

Disease site	Article	Number of Patients	Adapted Fractions Evaluated	Online ART procedural time	Outcomes
Bladder	Azzarouali et al. [43]	5	–	Median 32 min	Improved PTV coverage with adaptive planning
Bladder	Storm et al. [44]	17	132	Median 14 min	Intra-fractional variations during online ART of bladder cancer were limited, which may be explained by a strict bladder filling regimen
Bladder	Zwart et al. [61]	3	–	–	Adapted plan coverage was ≥ 99% for all sessions, compared to only 2/73 session reached this level for scheduled plans
Bony Metastases (Lumbar and Thoracic Spine, and Pelvis)	Nelissen et al. [57]	8	–	Average 36 min	Patients were satisfied with the procedure and completed consultation and treatment within two hours
Brain	Kang et al. [49]	7	–	Average 44.2 min*	Adaptation improved target coverage and limited hotspots in the hippocampal avoidance zone
Breast	Stanley et al. [58]	2	–	–	Daily adaptive replanning shows potential for reduced PTV margins and reduced OAR doses
Head and Neck	Dohopolski et al. [60]	10	–	–	Adapted planning significantly improved median V100% coverage, homogeneity, and total median dose reduction in OARs
Lung	Gonzalez et al. [46]	18	68	Average 15 min	Significant improved target coverage, dose conformity, and OAR sparing with online adaptive planning
Lower lung and Upper Abdomen	Kim et al. [50]	8	36	Average 27 min	CBCT-guide ART demonstrated inter- and intra-fractional motion Residual motion of tumor was comparable to that of the imaging-surrogate within clinical PTV margins (5 mm) but a bit larger than the pre-configured gating window
Liver, Pelvis, Abdomen, and Lung	Musunuru et al. [62]	15	–	–	Adapted plans had superior coverage, and nearly always met OAR tolerances compared to scheduled plans

*The initial adaptation is from a plan generated from a diagnostic image, not a sim CT

In head and neck cancers, a combination of factors such as tumor response, inflammation, muscle atrophy, and weight changes could alter target volumes and shift OARs into radiation fields over the course of a treatment [53]. An average of 20 min is spent on the Ethos™ ART process for head and neck malignancies [53]. For adaptive stereotactic partial breast irradiation (ABPI), the average ART procedural time was 15 min [54]. ART has also been used in hippocampal-avoidance whole brain radiotherapy (HA-WBRT) and has reported an average procedural time of 44 min by Kang et al., with approximately 22 min dedicated to contour adjustments for positional differences between diagnostic and on-table imaging [55]. It is also important to note that adaptation procedural time shortens with site experience and across subsequent fractions in individual patients [47, 49, 56].

Patients undergoing ART currently spend a significantly longer time on the couch during treatment than patients treated without on-table adaptation, with most of the time used for contouring edits and treatment plan review. Nevertheless, most ART sessions can fit into a normal clinical workflow without causing significant delay, as the majority of cases require minimal contouring edits and plan review. Given the potential improvement in target coverage and OAR sparing, the extended treatment time may be worthwhile. With the current state of rapidly improving technology in treatment planning and auto-segmentation [57], it is expected that ART procedural time will continue to decrease. In addition, sites involved in on-table ART have demonstrated that radiation therapists can lead the online ART workflow with minimal input from radiation oncologists, and achieve better efficiency with similar treatment efficacy [58].

### Treatment planning workflow

Multiple studies have shown that the treatment plan quality generated by the Intelligent Optimization Engine (IOE) is consistent with that of manually optimized treatment plans. Calmels et al., demonstrated that highly consistent IMRT plans were generated by the IOE in 60 pelvic cases with equivalent coverage and OAR sparing compared to manually optimized plans [36]. Roover et al., observed that the automated treatment plans for prostate SBRT cases achieves similar plan quality as those that were manually optimized, citing inter-planner variation as the main cause of dosimetric differences [53].

The Ethos™ automated treatment planning process differs from the traditional steps that are utilized in conventional treatment planning. The most notable difference comes from the automated optimization process that is conducted by the IOE. The IOE takes the input of an ordered and ranked list of clinical goals as the physician’s intent. This intent is converted into traditional optimization objectives with additional heuristics and optimization structures the user does not control [29]. Careful consideration of these ranked and ordered goals are important as they are also used for generating the online adaptive treatment plans which account for dosimetric tradeoffs that can be seen in the patient anatomy of the day. The process of optimizing the plans via clinical goals allows clinicians to make intuitive judgments when comparing the dosimetric results between the scheduled and adapted plans.

The Ethos™ treatment unit utilizes Mobius 3D-Adapt as an independent plan QA check for online adaptive radiation therapy treatments. A retrospective study by Zhao et al. performed patient specific QA for 16 adaptive plan sessions and found the gamma passing rate to be 99% (± 0.7%) [59].

### Auto-contouring impact on plan quality

High-quality, high-efficiency ART sessions require a robust auto-contouring and auto-planning system. In a study of 25 prostate cancer patients, the quality of Ethos™ autocontours before and after manual editing were studied [60]. Moazzezi et al. reported that for most patients even without manual edits, the target coverage and OAR doses met clinical goals after adaption [60]. Mao et al. in a study of 10 locally advanced lung patients (and 290 total fractions), also compared the dosimetric consequences of adapting with unedited vs. edited autocontours [61]. They found that clinical target volume (CTV) coverage was improved by adapting with autocontouring, while further improvements were made with manually edited structures. OAR doses were sometimes decreased but not cumulatively across the treatment course. They concluded that “Accuracy of Ethos™ automatic contouring is considered clinically acceptable”. Byrne et al. also reported that the adaptive plan was selected in 95% of the delivered fractions over the scheduled plan; 11% of the auto-generated contours needed no changes and 81% required only minor edits [44]. Nevertheless, physician review of daily auto-segmentation was still necessary for all patients in the case of outliers [60].

The consistency of Ethos™ auto-contouring and auto-planning functionalities has been positively reported in the literature. Chapman et al. used deformations of a pelvic phantom to evaluate the robustness and reproducibility of Ethos™ auto-segmentation and planning [57]. High reproducibility and accuracy were observed for structures such as femoral heads, bowel, and rectum; reproducibility was less consistent for prostate and bladder, both of which more often required additional editing [57]. Despite the large deformations in the target and surrounding OARs, auto-generated plans met all clinical constraints [57]. Limitations with regard to auto-contouring can occasionally provide dosimetrically less accurate plans. For example, air-gaps between bolus and skin are often filled in during auto-contouring and assumed to be excess tissue unless corrected by user, or large changes in bowel gas leading to inaccurate deformations [62].

### Ethos-driven single visit palliative treatment

In palliative treatment, a fast workflow is ideal to hasten relief, reduce anxiety, and minimize inconvenience for the patient. In one study of bony metastases in the spine and pelvis, 47 patients were treated in a single visit without a planning CT scan [63]. A “rough” plan was generated based on diagnostic images prior to the visit, and the plan was then adapted online using the Ethos™ CBCT [63, 64]. Adaptative plans were selected for all patients because of significant improvements in target coverage (PTV/CTV V_95%_, *p* value < 0.005) compared to the diagnostic based non-clinical reference plan and the majority of patients (~ 63%) required no or only minor contouring edits [63]. The study met its main goal to implement a simulation free workflow for single visit delivery of CBCT-based ART delivery within 2 h, leveraging diagnostic CT for pre-planning; median time for the workflow was 85 min, with 30 min spent in the treatment room. Most importantly, patients reported satisfaction with length of the consultation and treatment session, with 80% of patients stating they would choose future radiation procedures in the same treatment pathway [63].

### OAR sparing and target coverage improvements

Over a course of treatment, normal physiologic organ shifts and radiation-induced tumor responses may lead to anatomical changes in the disease target and surrounding tissues. Significant dosimetric improvements are derived using Ethos™ CBCT-guided online ART. In abdominal oligiometastases, according to Schiff et al., 75% (30 out of 40) of the fractions had OAR constraint violations without plan adaption, while only 5% (2 out of 40) of the fractions had violations with adaptation [31]. Similar improvements were achieved in pancreatic cancer, in which 97.5% (39 out of 40) non-adapted fractions had OAR constraint violations, compared to 0% (0 out of 40) in adapted fractions [47]. OAR dose reductions were also seen with Ethos™ in breast [65], bladder [39], cervical [40, 51], prostate [42–44, 60, 66], and head and neck [67] malignancies.

In terms of target coverage, gross tumor volume (GTV) V_100%_ and D_95%_ improved in 62.5% (25 out of 40) and 50% (20 out of 40) of abdominal oligometastases plans with the use of Ethos™ CBCT-guided online ART [31]. Adaptive planning also achieved better planning target volume (PTV) coverage in studies involving diseases sites in breast [54, 65], bladder [39, 49, 68], brain [55], head and neck [53, 67], lung [52, 61], pancreas [47], pelvis [46, 69], and prostate [42–44, 66]. However, for cervical and rectal cancers, the reported target coverage was similar between adapted and non-adapted plans [40, 70]. In one study of prostate cancer by Moazzezi et al., the improvement in CTV D_98%_ with ART was minimal [60].

During treatment planning, narrower margins help reduced OAR doses while preserving target coverage. Ethos™ CBCT-guided ART accounts for interfraction changes and, due to the treatment speed, minimizes the impact of intrafraction motions allowing for margin reductions. *Ray *et al*.* reported that prostate margins could be reduced to 3 to 4 mm symmetrically without altering the CTV coverage with the use of Ethos™ [71]. In treated bladder cancer, ART plans achieved a median 42% primary PTV reduction and 24–30% V_45Gy_ reduction to the bowel cavity compared to non-ART plans [46]. Significant PTV bladder and head and neck volume reduction were also achieved by Aström et al. [39] and Dohopolski et al. [67], respectively.

### Motion management

Target and OAR motion during the ART procedure may result in errors and reduce the quality of adaptive plans. Surface-guided radiation therapy (SGRT), which uses optical surface scanning for patient positioning, can be used with ART for intra-fraction motion monitoring and respiratory gating especially for lung and abdominal malignancies [72]. It provides real-time motion monitoring of the patient surface throughout the whole treatment fraction. The beam can be held if parts of the patient’s surface deviate from the reference position based on the planning CT set-up or if the calculated isocentric deviations exceed a certain threshold [72].

Nevertheless, the actual magnitude of internal organ movement during procedure treatment was previously unknown. Storm et al. studied intra-fraction bladder motion during the adaptive procedure in 17 patients treated with Ethos™ by comparing the patient positions from the initial CBCT, prior to the ART process, to the CBCT directly before beam on time and observed only small changes in bladder volume and center of mass position, with a median time of 14 mintues between the two scans [50]. Zwart et al. compared prostate position on the CBCT taken just prior to the beam on time to that on the CBCT immediately after treatment, with a mean time of 4.2 ± 0.6 min between scans [73]. The 95th percentile of prostate motion ranged from 1.7 mm in the x-direction to 3.2 mm in the z-direction, which suggests that smaller PTV margins can be safely implemented in clinical practice. Storm et al. captured a median 8.5 cm^3^ increase in bladder filling volume with a second CBCT after the adaptive planning CBCT and prior to treatment delivery [50]. Finally, Jong et al. also obtained a second CBCT to validate with respect to the CTV coverage immediately prior to treatment delivery, which took on average 20 min, and reported excellent motion management in rectal cancer in the vast majority of cases except for two incidence of workflow interruption due to the second CBCT having insufficient target coverage [26].

### Future directions

The studies summarized in this review have shown improvements in radiotherapy by reducing doses to normal tissues, improving target coverage, and increasing the potential for dose escalation. However, the question that naturally follows is, do these technical advances translate into improved clinical outcomes? Currently, there are ongoing clinical trials investigating clinical outcomes and PROs obtained with the use of Ethos™ CBCT-based ART across a wide range of disease sites including cancers of the head and neck (NCT04883281, NCT04379505), lung (NCT05488626), pancreas (NCT05764720), bladder (NCT05295992, NCT05700227), cervix (NCT05197881), and anus (NCT05438836). These studies are designed to demonstrate meaningful improvements in treatment-related side effects, such as acute GI toxicity in the treatment of bladder cancer and advanced cervical cancers, by comparing Ethos™ CBCT-based ART to the standard of care.

## Conclusion

Online ART entailing treatment plans adjusted prior to delivery to account for temporal and stochastic changes observed in a single treatment fraction while the patient remains in the treatment position is feasible with Ethos™ CBCT-guided ART system. The auto-segmentation tool performs well with some editing and the procedural time with learning can be reduced to 15–30 min based on the complexity of anatomical site. There are dosimetric gains seen in either reduced dose to OARs, improved target coverage, or dose escalation while maintaining OAR tolerance doses. Ongoing prospective studies will help define clinical gain in terms of local control, reduced morbidities, and better patent-reported outcomes.

## Data Availability

Research data is stored in Varian repository and will be shared upon request to the corresponding author.

## Abbreviations

CT: Computed tomography
CBCT: Cone beam computed tomography
iCBCT: Iterative cone beam computed tomography
ART: Adaptive radiotherapy
OAR: Organs-at-risk
kV: Kilovoltage
QA: Quality assurance
IGRT: Image-guided radiotherapy
AI: Artificial Intelligence
MRI: Magnetic Resonance Imaging
FDA: Food and Drug Administration
IMRT: Intensity-modulated radiotherapy
VMAT: Volumetric modulated arc therapy
SBRT: Stereotactic body radiotherapy
GI: Gastrointestinal tract
LAPC: Locally advanced pancreatic cancer
ABPI: Adaptive stereotactic partial breast irradiation
HA-WBRT: Hippocampal-avoidance whole brain radiotherapy
IOE: Intelligent optimization Engine
CTV: Clinical target volume
PTV: Planning target volume
GTV: Gross tumor volume
SGRT: Surface-guided radiation therapy
